# Biowaste derived hydroxyapatite embedded on two-dimensional g-C_3_N_4_ nanosheets for degradation of hazardous dye and pharmacological drug via Z-scheme charge transfer

**DOI:** 10.1038/s41598-022-15799-y

**Published:** 2022-07-07

**Authors:** Palanisamy Govindasamy, Bhuvaneswari Kandasamy, Pazhanivel Thangavelu, Selvaraj Barathi, Maiyalagan Thandavarayan, Mohd. Shkir, Jintae Lee

**Affiliations:** 1 School of Chemical Engineering, Yeungnam University, Gyeongsan, Gyeongbuk 38541 Republic of Korea; 2 Department of Electronics and Communication Engineering, Sri Sivasubramaniya Nadar College of Engineering, Tamil Nadu, Kalavakkam, 603 110 India; 3 Smart Materials Interface Laboratory, Department of Physics, Periyar University, Tamil Nadu, Salem, 636 011 India; 4 Department of Chemistry, SRM Institute of Science and Technology, Kattankulathur, 603203 Tamil Nadu India; 5 Advanced Functional Materials and Optoelectronics Laboratory (AFMOL), Department of Physics, Faculty of Science, King Khalid University, Abha, 61413 Saudi Arabia; 6 Department of Chemistry and University Centre for Research and Development, Chandigarh University, Mohali, 140413 Punjab India

**Keywords:** Environmental sciences, Materials science, Nanoscience and technology

## Abstract

In recent years, there has been an increase in demand for inexpensive biowaste-derived photocatalysts for the degradation of hazardous dyes and pharmacological drugs. Here, we developed eggshell derived hydroxyapatite nanoparticles entrenched on two-dimensional g-C_3_N_4_ nanosheets. The structural, morphological and photophysical behavior of the materials is confirmed through various analytical techniques. The photocatalytic performance of the highly efficient HAp/gC_3_N_4_ photocatalyst is evaluated against methylene blue (MB) and doxycycline drug contaminates under UV–visible light exposure. The HAp/gC_3_N_4_ photocatalyst exhibit excellent photocatalytic performance for MB dye (93.69%) and doxycycline drug (83.08%) compared to bare HAp and g-C_3_N_4_ nanosheets. The ultimate point to note is that the HAp/gC_3_N_4_ photocatalyst was recycled in four consecutive cycles without any degradation performance. Superoxide radicals play an important role in degradation performance, which has been confirmed by scavenger experiments. Therefore, the biowaste-derived HAp combined with gC_3_N_4_ nanosheets is a promising photocatalyst for the degradation of hazardous dyes and pharmacological drug wastes.

## Introduction

In recent decades, the dyeing process is mainly used in the manufacture of cosmetics, textiles, tints, and medical products. During the dyeing process, around 15% of the untreated dyes are directly released into the environment, which is highly toxic to the ecosystem^1^. Generally, the dyes are highly stable and can’t degrade in routine cycle^2^. Therefore, it has become very important in recent years to develop treatment methods for removing dyes that can be easily removed from natural water bodies. On the other hand, the usage of drugs and antibiotics is widespread in the treatment of infections in humans and other living organisms. Various antibiotics are commonly used in the infections, such as ciprofloxacin^3^, azithromycin^4^, erythromycin^5^, ofloxacin^6^, doxycycline^7^, etc. Due to incomplete metabolism, remaining antibiotics are released back into the environment after the treatment process and form the superbugs in the human body. Among various antibiotics, doxycycline is a semi-synthetic tetracycline antibiotic and it is extensively used to treat diseases in living organisms. Doxycycline drug is non-biodegradable, so the complete degradation of doxycycline is necessary for a healthy society. In this connection, several conventional degradation methods like adsorption^8^, ion flotation^9^, filtration^10^, coagulation^11^ and solvent extraction^12^ are extensively used to degrade the various organic dyes and doxycycline drugs. These conventional approaches only transform organic pollutants from a liquid phase to a solid phase, they produce secondary hazardous contamination. Among these approaches, semiconductor photocatalysis methods are an effective technology for breaking down dangerous dye molecules in a short time. In addition, semiconductor photocatalysis techniques exhibit peculiar properties like low-cost, high efficiency, and non-corrosive properties. Generally, the efficient photocatalyst will degrade organic contaminants in an aqueous system in a stable, cost-effective, non-toxic, and visible light-active manner.

Semiconductor metal oxide materials are a promising photocatalyst due to their excellent photonic stability and physicochemical properties. The conventional materials (TiO_2_ and ZnO) are only active in ultraviolet light due to the large energy bandgap and rapid electron–hole recombination. To overcome these phenomena, our current research focuses primarily on the development of eco-friendly calcium hydroxyapatite-based photocatalysts. Hydroxyapatite (Ca_10_(PO_4_)_6_(OH)-HAp) is one of the major materials for bone and teeth. Several of its unique properties, including its nontoxic nature, bioactivity, and biocompatibility, have made it useful in orthopedic treatments. On other hand, its high specific surface area, low solubility in water, mechanical stability and high thermal stability makes it an excellent green photocatalyst for the decomposition of hazardous dye molecules. For example, Jie Yao et.al^13^ have reported that the hydroxyapatite nanoparticles combined with TiO_2_ nanoparticles improve the decontaminate removal of nitric oxide in the air under simulated solar light irradiation. Furthermore, the presence of phosphate groups in the hydroxyapatite materials creates more superoxide radicals during the photocatalytic activity. Nishikawa et al.^14^ report that oxygen vacancies on the surface of hydroxyapatite materials act as an electron-acceptor in the photocatalytic process. Generally, hydroxyapatite does not degrade organic dyes under visible light due to its large energy bandgap. To address this issue, we are planning to combine visible active materials in hydroxyapatite to enhance performance in visible light while decreasing degradation time. Chang et al.^15^ have reported carbon dot-supported hydroxyapatite exhibit superior photocatalytic performance of MB dye degradation under visible light irradiation. Recently, a variety of semiconductor materials like TiO_2_^13^, Ag_3_PO_4_^16^, Fe_3_O_4_^17^, CoFe_2_O_4_^18^, g-C_3_N_4_^19^, rGO^20^, etc. have been studied to enhance hydroxyapatite's performance. However, chemically synthesized hydroxyapatites are more expensive due to the calcium and phosphate sources^21^. Currently, researchers are focusing a great deal of attention on biogenetic wastes such as mussel shells, oyster shells, fish scales, biomass ashes, snail shells, eggshells, etc., being used as a means to reduce costs and recycle waste to make value-added materials^22^. Comparatively to other derived HAp, eggshell-derived HAp has received a lot of attention in recent years.

On the other hand, two-dimensional g-C_3_N_4_ nanosheets exhibit outstanding photocatalyst materials due to their high thermal stability, high surface area, suitable energy bandgap, enhanced electron–phonon interaction and eco-friendly material. These peculiar properties combine with HAp nanoparticles to enhance decomposition performance. For example, Mohammad Chahkandi et al.^19^ have prepared HAp nanosphere decorated two-dimensional g-C_3_N_4_ nanosheets that exhibit excellent photocatalytic degradation of bisphenol A under visible light irradiation. On the other hand, Tianhong Xu et al.^23^ reported the presence of strong and highly potent g-C_3_N_4_/HAp composites using decomposed tetracycline drugs under UV–visible light. Consequently, the two-dimensional g-C_3_N_4_ nanosheet enhances the active sites and promotes degradation of hazardous dye molecules and pharmacological waste.

Based on the above problems, we developed eggshell derived small-sized HAp nanoparticles that adorn the surface of gC_3_N_4_ nanosheets using an in-situ precipitation method to make a Z-scheme based green photocatalyst with high catalytic performance and photostability. In order to characterize the physicochemical and morphological properties of the prepared samples analyzed through various characterization techniques. The degradation performance of the Z-scheme based HAp/gC_3_N_4_ nanocomposites was evaluated against methylene blue (MB) dye and doxycycline drug under UV–visible light irradiation. The scavenger test was used to confirm and explain the full details of the possible photocatalytic mechanism. This study recommends that eggshell derived Z-Scheme HAp/gC_3_N_4_ nanocomposites will be an effective photocatalyst in wastewater treatment applications.

## Experimental procedure

### Chemicals and reagents

Reagents used for tests are di-ammonium hydrogen phosphate [(NH_4_)_2_HPO_4_], nitric acid [HNO_3_], ammonia solution [NH_4_OH], sulfuric acid (H_2_SO_4_) and Melamine (C_3_H_6_N_6_) were purchased from Merck and SDFCL, India. No additional purification was performed on any of the reagents. CaO nanopowder used for the experiment was obtained by heating eggshells at 900 °C.

### Preparation of g-C_3_N_4_ nanosheets

The thermal condensation technique was used to prepare graphite-like C_3_N_4_ sheets. In an aluminium crucible, the melamine powder was placed and then heated at 560 °C for four hours in a muffle furnace. The obtained g-C_3_N_4_ samples were crushed to a fine powder after cooling down to room temperature. The g-C_3_N_4_ nanopowder was pre-treated with sulfuric acid at room temperature for five hours prior to protonation. The protonated g-C_3_N_4_ nanopowder was then rinsed with deionized water to the neutral condition and then dried at 90 °C overnight.

### Preparation of eggshell derived HAp and HAp/gC_3_N_4_ nanocomposites

**Figure 1:**
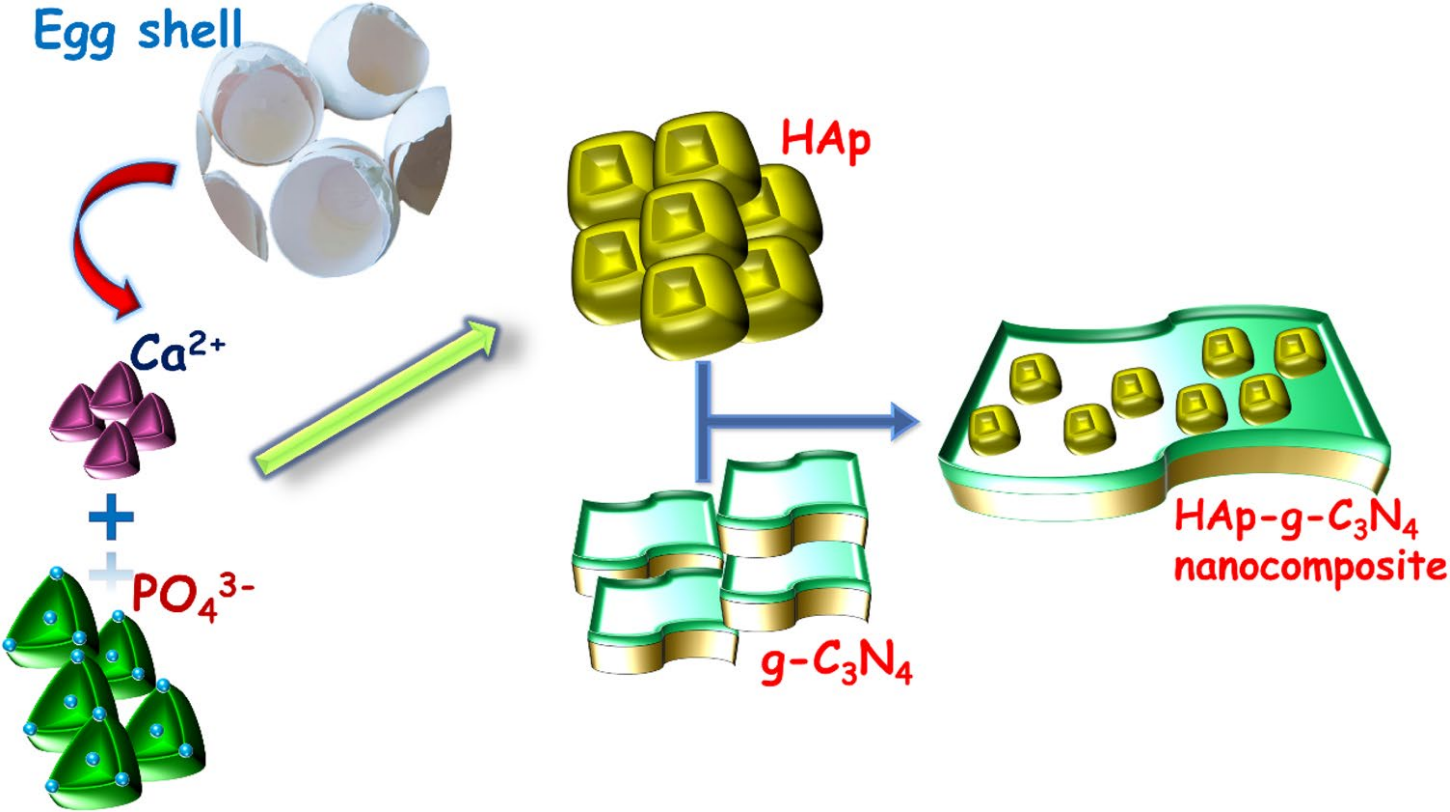
Schematic representation of the synthesis of HAp/gC_3_N_4_ nanocomposites.

HAp/gC_3_N_4_ nanocomposite was prepared by an in-situ precipitation method. Briefly, a batch of eggshells was collected in large quantities. Deionized water and boiling water for 30 min were used to manually clean them. Afterward, cleaned eggshells were placed in a muffle furnace at 900 °C for a 60 min of the thermal treatment process. When the eggshells reached 900 °C, they converted into CaO and released CO_2_. Further, 0.1 mol/L of calcium oxide was dissolved in nitric acid to produce calcium nitrate**.** With vigorous stirring at room temperature, 0.06 mol/L of (NH_4_)_2_HPO_4_ solution was added dropwise to the calcium nitrate solution. An additional 1.5 g of g-C_3_N_4_ nanosheets was added to the prepared reaction mixture after it had been stirred for one hour and aged for 1 day at room temperature. To separate the precipitate from the by-products, the mixture was centrifuged and then rinsed several times. Afterward, the precipitate was dried in a hot air oven for 6 h at 120 °C, and the obtained solid powder was crushed into the fine powders. A similar process was carried out in the absence of g-C_3_N_4_ to obtain pure HAp samples for comparison. The preparation of HAp/gC_3_N_4_ nanocomposites was illustrated in Fig. 1.

### Materials characterization

The structure and sample purity of the as prepared HAp, gC_3_N_4_ and HAp/gC_3_N_4_ nanocomposites were analyzed by XRD powder diffraction (XRD), and for this a Rigaku MiniFlux-II X-ray diffractometer. The binding energies of HAp/gC_3_N_4_ nanocomposites were analyzed through XPS analysis (PHI-VERSAPROBE III spectroscopy). The shape, detailed morphology and chemical composition of the synthesized photocatalysts were studied using a Field emission scanning electron microscope, energy dispersive X-ray spectrometer (Quanta FEG 250) and High-resolution transmission electron microscopy (JEOL-2100 + electron microscope). An infrared (FTIR) spectrometer model Bruker Tensor 27 was used to study the interaction between HAp and gC_3_N_4_. A SHIMADZU 3600 UV–Vis-NIR model spectrophotometer was used to obtain optical absorption spectra. Horiba Jobin Yvon Spectro Fluromax 4 was used to measure emission spectra.

### Photocatalytic activity evaluation

To assess the photocatalytic effectiveness of the as-prepared photocatalysts, the photocatalytic degradation of MB dye and doxycycline drug was examined under irradiation with UV–visible light. A 500 W tungsten-halogen lamp was used to provide UV–visible light irradiation. In a typical reaction, the dye concentration was set to 30 ppm, and a known quantity of photocatalyst (0.050 g) was added to the dye mixture. Before starting the irradiation, the dye mixture was stirred and placed in a dark place to found adsorption–desorption equilibrium. The reaction vessel has an outer jacket for water circulation to keep the reaction mixture at ambient temperature. During irradiation, 3 mL of dye solution was kept at every 20 min. breaks and the photocatalyst was removed by the centrifugation process. A UV–Vis spectrophotometer was used to measure the concentration of MB and doxycycline drug.

### Detection of active species

In order to identify which radical species are a major role in the photocatalytic process using the scavenger test. The scavenger experiment was similar to the photocatalytic activity evaluation. Different scavengers like Ethylenediaminetetraacetic acid (EDTA), isopropyl alcohol (IPA), and 1,4-benzoquinone (BQ) were added to detect the holes, hydroxyl, superoxide radical in the doxycycline and organic dye solution before the light irradiation.

## Results and discussion

**Figure 2:**
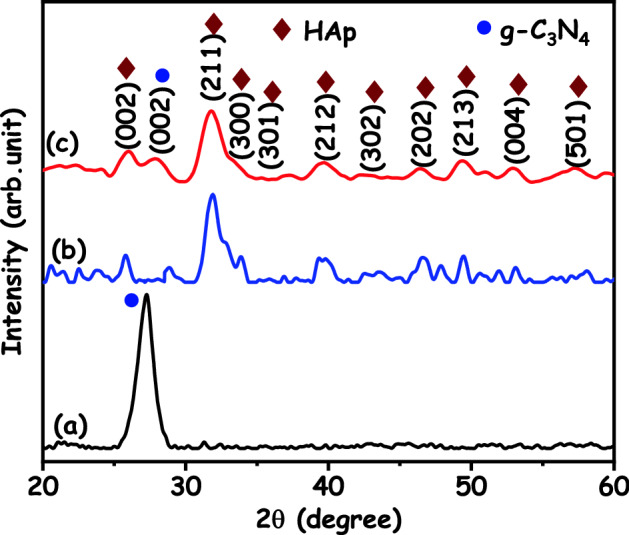
X-ray diffraction patterns of (**a**) g-C_3_N_4_, (**b**) HAp and (**c**) HAp/g-C_3_N_4_ nanocomposites.

Figure 2 shows the XRD patterns of g-C_3_N_4_, HAp and HAp/g-C_3_N_4_ nanocomposites. The most evident diffraction peak is found at 2θ = 27.2° and 13.0° in the g-C_3_N_4_ were indexed to the (002) and (100) diffraction planes, respectively. These diffraction planes are in good agreement with the standard hexagonal graphitic carbon nitride (JCPDS file no. 87-1526)^24^. For the pure HAp (Fig. 2b), the diffraction peaks of 25.6°, 31.8°, 33.8°, 35.3°, 39.3°, 42.5°, 46.3°, 49.3°, 52.8° and 57.6° were ascribed to the respective (002), (211), (300), (301), (212), (302), (202), (213), (004) and (501) diffraction planes of the hexagonal phase (JCPDS, 09-0432)^25^. The HAp/g-C_3_N_4_ nanocomposites show diffraction peaks for both HAp and g-C_3_N_4_. Furthermore, the intensity of the diffraction peak (211) was increased when g-C_3_N_4_ nanosheet was added to HAp nanoparticles. It is clearly indicated that the presence of all diffraction peaks confirms the effective formation of HAp/g-C_3_N_4_ nanocomposites. The Scherer’s equation is used to calculate the crystalline size of the as-prepared samples^26^. The calculated crystalline size of the g-C_3_N_4_, HAp and (211) planes of HAp/g-C_3_N_4_ nanocomposites are 15.46, 17.03 and 17.24 nm, respectively.

**Figure 3:**
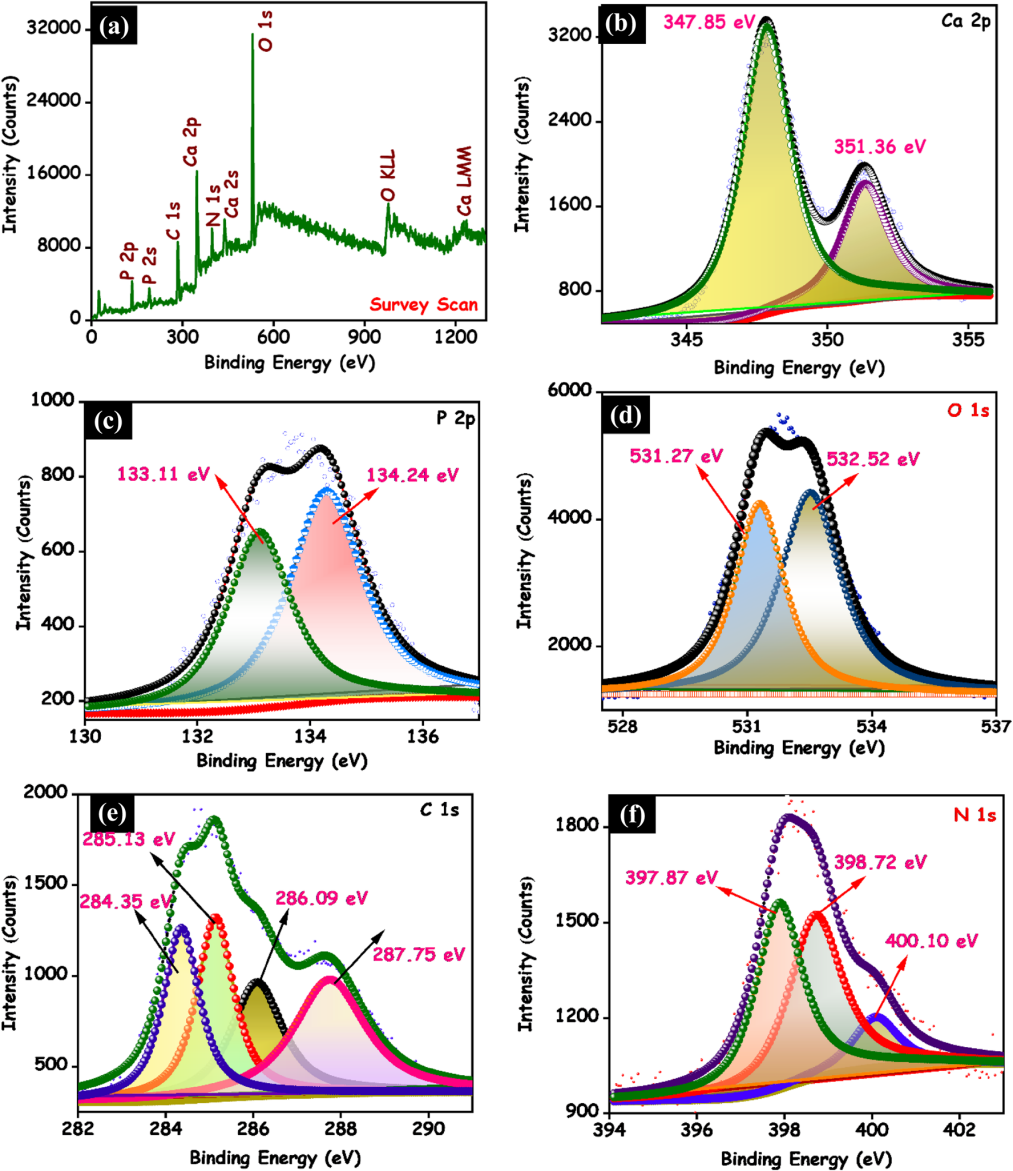
XPS spectra of g-C_3_N_4_/HAp nanocomposite. (**a**) Survey spectra, (**b**) Ca 2p, (**c**) P 2p, (**d**) O 1s, (**e**) C 1s and (**f**) N 1s.

The surface chemical composite and bond configuration of the as-prepared eggshell derived g-C_3_N_4_/HAp nanocomposite was recorded by X-ray photoelectron spectrum and the corresponding outcomes are shown in Fig. 3. In survey spectrum of g-C_3_N_4_/HAp nanocomposite (Fig. 3a) demonstrates the only existence of C 1s, N 1s, Ca 2p, P 2p and O 1s elements. Figure 3b displayed the high-resolution XPS spectra of Ca 2p can be deconvoluted into two peaks at 347.5 and 351.36 eV, which might be attributed to the Ca 2p_3/2_ and Ca 2p_1/2_ of Ca^2+^ oxidation state in the hydroxyapatite respectively. These results are similar to those reported in the previous article^19^. The P 2p spectra of g-C_3_N_4_/HAp nanocomposite was shown in Fig. 3c. The P 2p_3/2_ and P 2p_1/2_ peaks for HAp were ascribed at 133.11 and 134.24 eV, respectively. These binding energies are attributed to the existence of the Ca-P bonds in the hydroxyapatite.^23^. In Fig. 3d, the high-resolution O 1s spectrum exhibits two peaks of oxygen state including C–O (531.27) and C=O (532.54 eV), which is similar to the previously reported literature^19^.

In Fig. 3e, the C 1s spectrum of g-C_3_N_4_/HAp nanocomposite can be deconvoluted into four peaks 284.35, 285.13, 286.09 and 287.75 eV, which are ascribed to the surface adventitious carbon C–C bonds, sp^3^-coordinated carbon bonds (C=O), carbon–nitrogen bond (C–N), and sp^2^-bonded carbon (N–C=N) in the tri-s-triazine rings, respectively. The high-resolution N 1s spectrum could be fitted into three peaks centered at 397.87, 398.72 eV and 400.10 eV. These peaks are attributed to the graphitic nitrogen (C–N), triazine rings (C–N–C) and tertiary nitrogen (N–(C)^3^) respectively (Fig. 3f). These values agreed with the prior literature values for the binding energies of nitrogen in g-C_3_N_4_ nanosheets^27^. Based on the XRD and XPS outcomes, it is clearly confirmed that g-C_3_N_4_/HAp nanocomposite is composed of g-C_3_N_4_ nanosheets and HAp.

**Figure 4:**
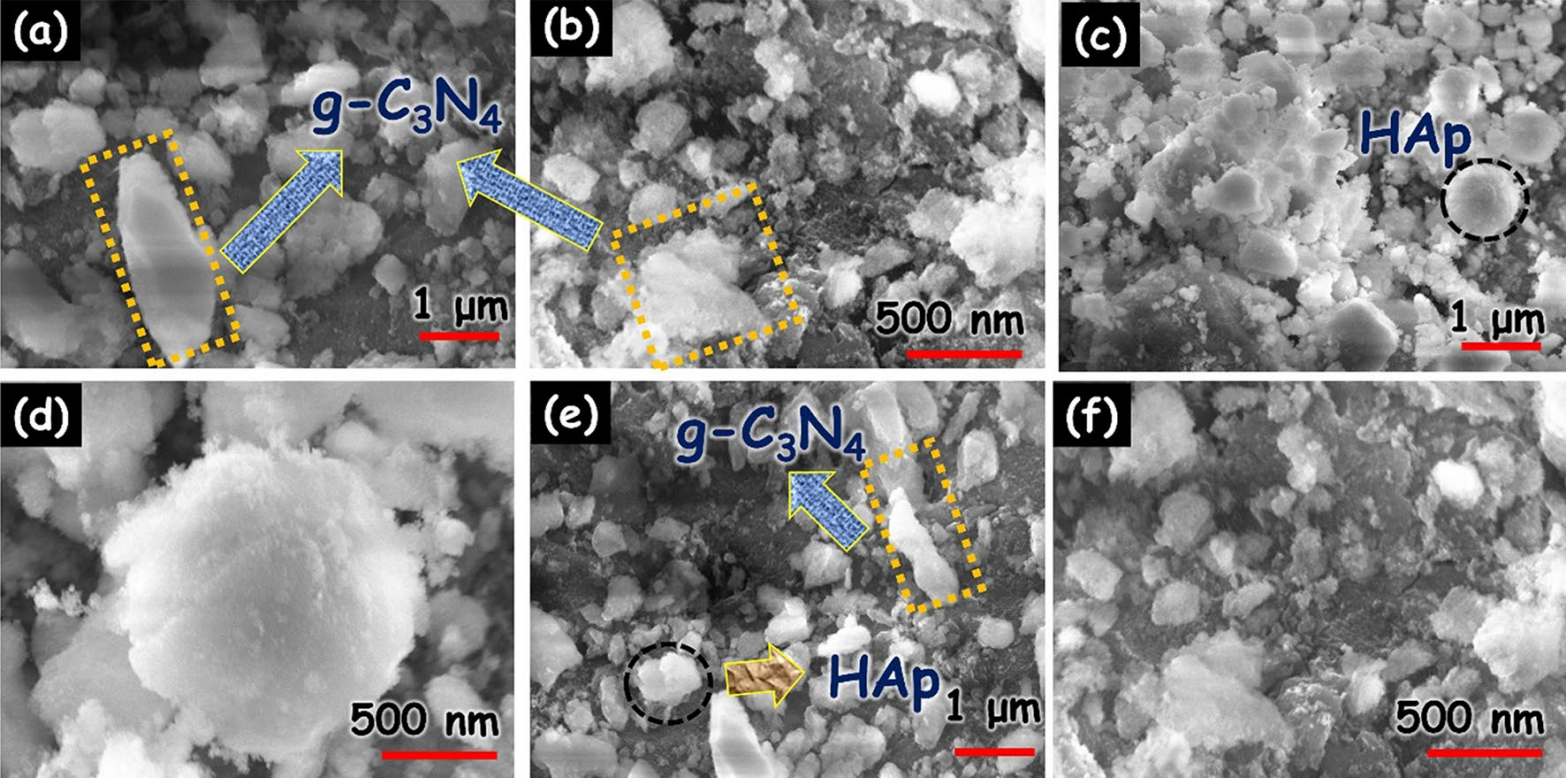
SEM image of (**a**,**b**) g-C_3_N_4_, (**c**,**d**) HAp and (**e**,**f**) HAp/g-C_3_N_4_ nanocomposites.

The shape and morphology of the prepared constituents were evaluated through SEM analysis and the gained consequences are exposed in Fig. 4. Figure 4a,b clearly shows the highly aggregated g-C_3_N_4_ wrinkled nanosheet with an irregular shape. In addition, the pure HAp (Fig. 4c,d) displays sphere-shaped nanoparticles with smooth surfaces. In the FESEM image of the HAp/g-C_3_N_4_ nanocomposites (Fig. 4e,f), it can be clearly shown that the small-sized HAp nanoparticles embedded on the surface of the g-C_3_N_4_ nanosheets.

**Figure 5:**
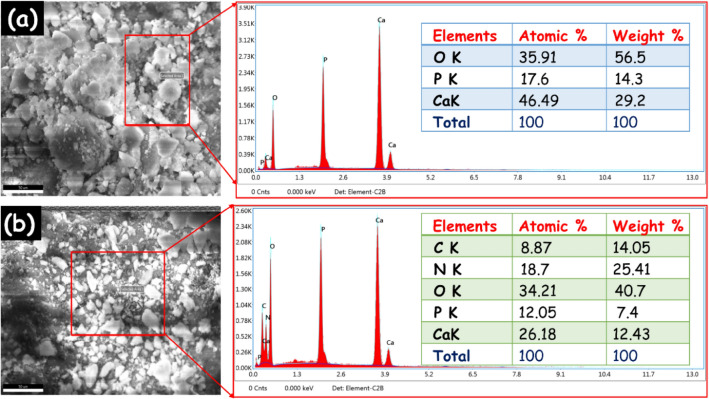
EDAX spectra of (**a**) HAp and (**b**) HAp/g-C_3_N_4_ nanocomposite.

**Figure 6:**
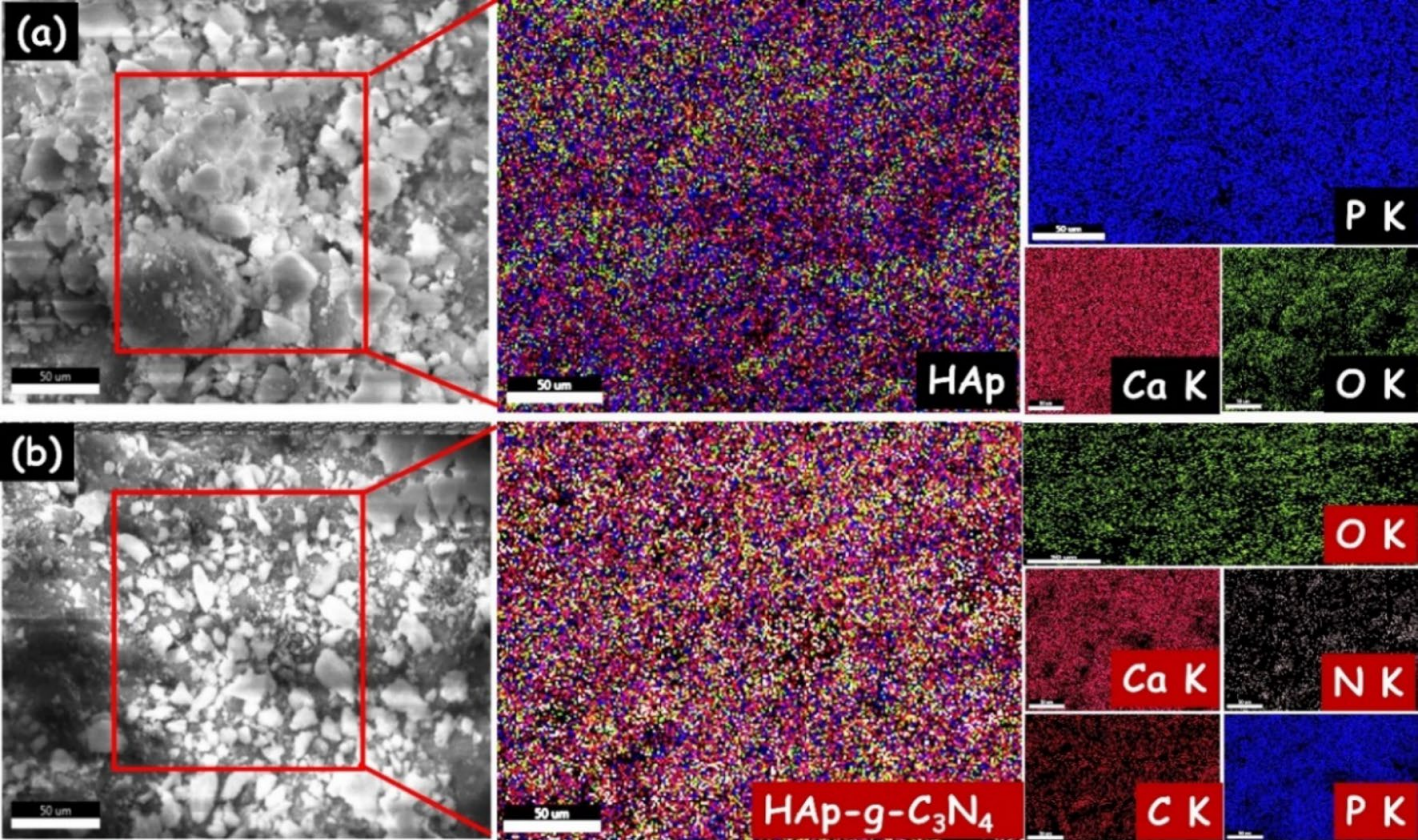
EDAX elemental mapping of (**a**) HAp and (**b**) HAp/g-C_3_N_4_ nanocomposite.

To further confirm the chemical composition of the prepared samples are analyzed through EDAX analysis. The EDAX consequences demonstrate only the existence of Ca, P and O elements (Fig. 5a). These findings clearly show that no other contaminants have been discovered. The EDAX image of the HAp/g-C_3_N_4_ nanocomposite (Fig. 5b) shows purely the presence of C, N, Ca, P, and O elements, indicating the materials are high purity level. The pure HAp and HAp/g-C_3_N_4_ nanocomposite's atomic and weight percentages are inserted into the relevant EDAX spectra. Additionally, the EDAX element mapping images of HAp/g-C_3_N_4_ nanocomposite demonstrate the existence of C, N, Ca, P and O elements (Fig. 6a,b). It is clear that the C, N, Ca, P and O are homogeneously distributed in the HAp/g-C_3_N_4_ nanocomposite, indicating the existence of g-C_3_N_4_ and HAp in the composite sample. The consequences were in good agreement with the analysis of XRD and XPS analysis.

**Figure 7:**
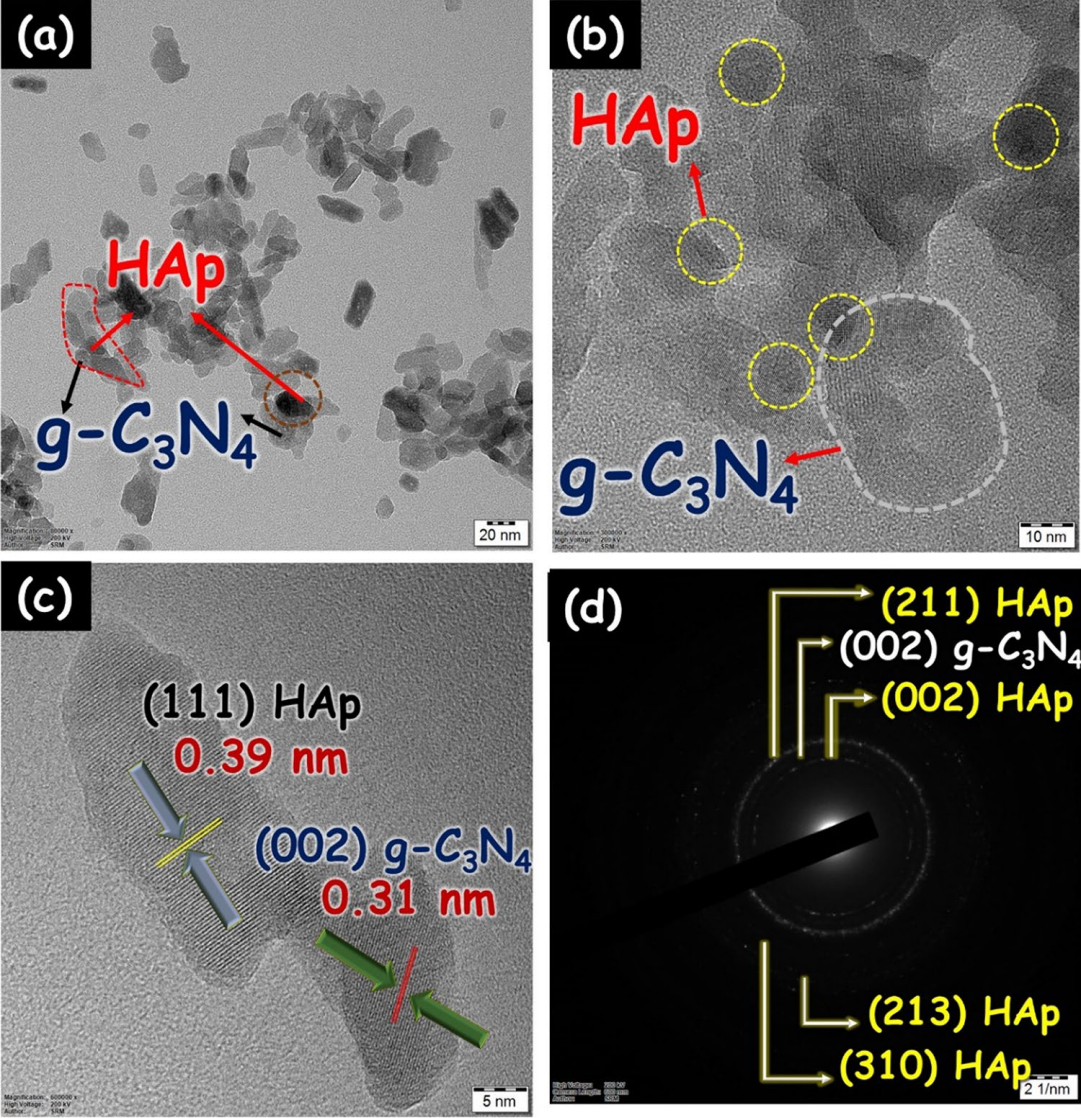
(**a**–**c**) HRTEM image and (**d**) SAED pattern of HAp/g-C_3_N_4_ nanocomposite.

To examine the detailed morphology of the HAp/g-C_3_N_4_ nanocomposite was examined through HRTEM analysis and the corresponding consequences are exposed in Fig. 7. Figure 7a–c clearly shows the small-sized nanosphere HAp was embedded on the two-dimensional g-C_3_N_4_ nanosheets. According to the lattice fringes in the HRTEM image (Fig. 7c), the resolved interplanar distance of 0.31 and 0.39 nm is consistent with the lattice spacing of the (002) and (111) plane of g-C_3_N_4_ and HAp, respectively. The selected area electron diffraction of the HAp/g-C_3_N_4_ nanocomposite is shown in Fig. 7d. ImageJ software was used to measure the diameter of rings within the SAED pattern. The diameter of rings is well-matched with the d-spacing and the corresponding results are indicating the effective formation of HAp/g-C_3_N_4_ nanocomposite.

**Figure 8:**
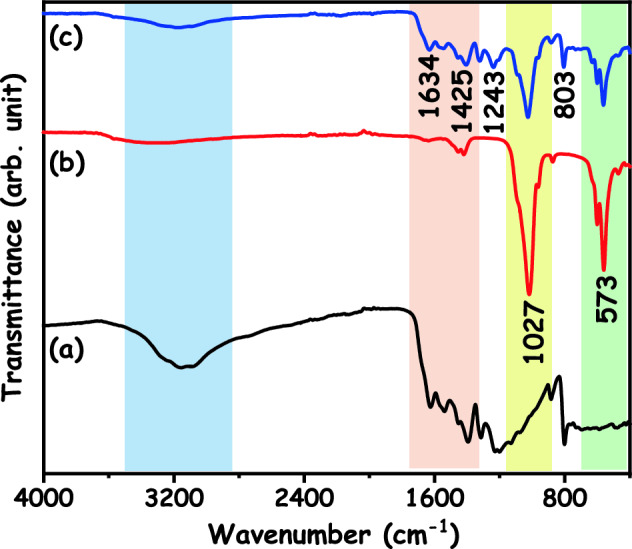
FTIR spectra of (a) g-C_3_N_4_, (b) HAp and (c) HAp/g-C_3_N_4_ nanocomposites.

The FTIR spectra of the g-C_3_N_4_, HAp and HAp/g-C_3_N_4_ nanocomposite are revealed in Fig. 8. As shown in Fig. 8a, the broad peak located at 3200 cm^−1^ are attributed to the N–H starching vibration modes. The peak in the range of 1650–1200 cm^−1^ are ascribed to the aromatic C–N and C=N hydro cyclic unit of g-C_3_N_4_. The sharp peak positioned at 807 cm^−1^ are ascribed to the breathing mode of the triazine units. In Fig. 8b, the characteristic broad peaks are observed in four different phosphate groups. In detail, the peak at 949 cm^−1^ is attributed to the symmetric P–O stretching vibrations (ν1) of phosphate groups. The bending vibration of the phosphate group (ν2) is located at 461 cm^−1^. The asymmetric P–O stretching (ν3) and bending (ν4) vibrations of the phosphate groups are located at 1027 and 573 cm^−1^, respectively^28^. When the combination of g-C_3_N_4_ and HAp, it can be the clear blue shift of the peak at 573 cm^−1^. In this connection, we propose a possible explanation for this phenomenon. Through the formation of the HAp/g-C_3_N_4_ nanocomposite, a partial electron transfer from g-C_3_N_4_ to HAp can improve the electron dispersibility of g-C_3_N_4_, thus refining the conjugation effect of g-C_3_N_4_. In Fig. 8c, the aromatic C=N vibration intensity is reduced and at the same time, they shift to higher wavenumbers. Based on these results, it is expected that g-C_3_N_4_ and HAp have an interfacial interaction that simplifies the transfer of photoinduced charge, thus refining the catalytic properties of HAp/g-C_3_N_4_ nanocomposite.

**Figure 9:**
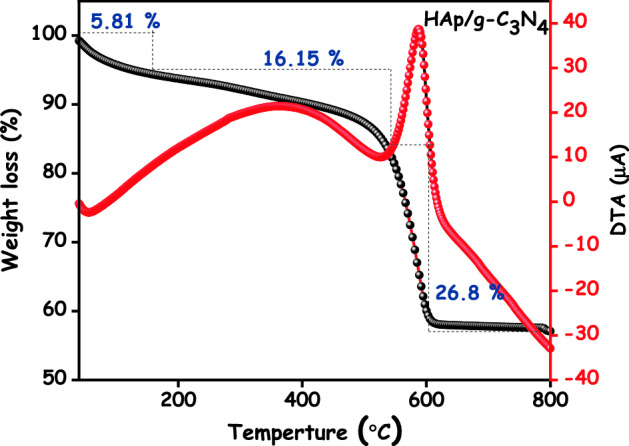
TG/DTA curve of HAp/g-C_3_N_4_ nanocomposites.

To determine the thermal stability of a HAp/g-C_3_N_4_ nanocomposite, Thermogravimetry and Differential Thermal Analysis **(**TG/DTA) were conducted and the corresponding outcomes are displayed in Fig. 9. TG/DTA curve of HAp/g-C_3_N_4_ nanocomposites was recorded between 36 and 800 °C at a heating rate of 20 °C/min. Since the HAp/g-C_3_N_4_ nanocomposite thermally decomposes, several steps occur and 58.28% of the residue appears according to the thermal analysis at 800 °C. When the temperature is raised to up to 150 °C, 5.81% of the weight decreases due to loss in adsorbed water molecules. Further heating of the sample to 320 °C resulted in a weight loss of 16.15% due to entrapped chemisorbed water. Due to the decomposition of the carbonate and other impurities, the third weight loss (26.8%) took place between 400 and 590 °C^29^. There has been no weight loss observed after 600 °C, which clearly indicates that the as prepared HAp/g-C_3_N_4_ nanocomposite has a high thermal and phase stability. In the DTA analysis, the two endothermic peaks at 120 °C and 587.5 °C are likely due to adsorbed water loss and other carbonate impurities, respectively.

**Figure 10:**
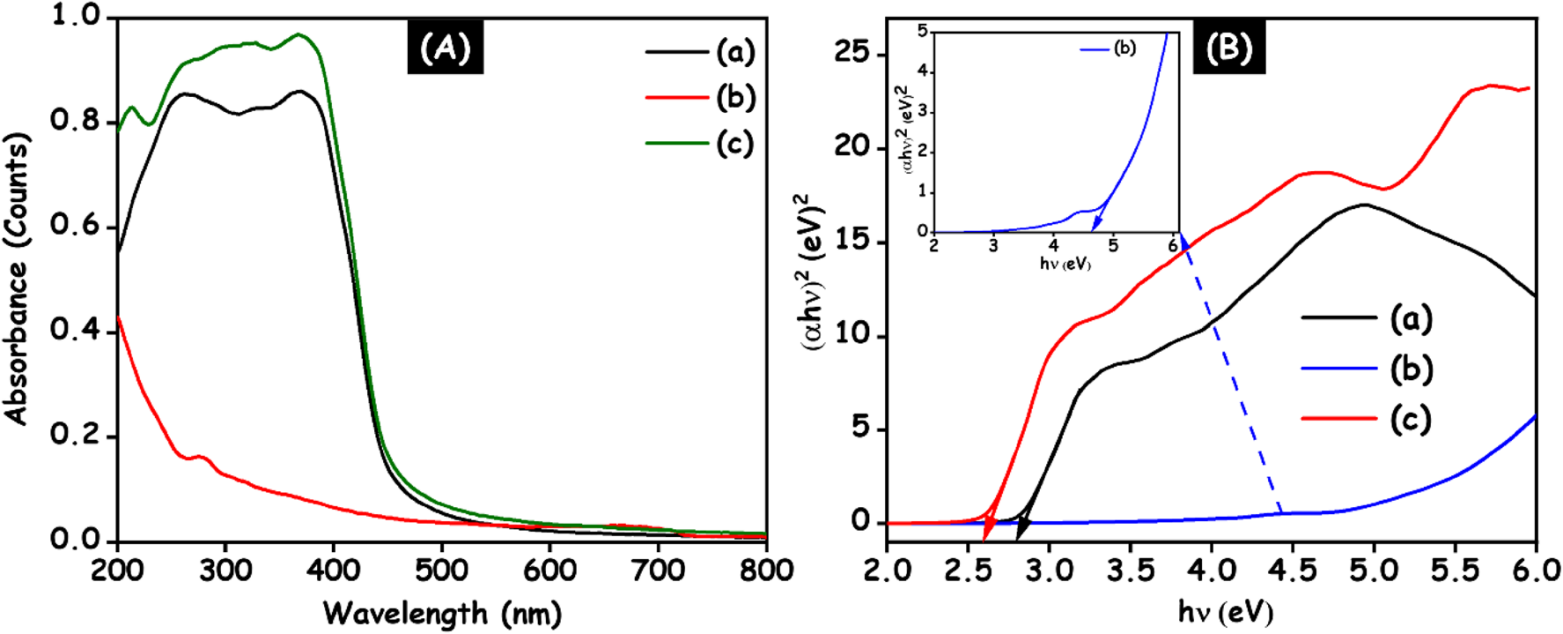
(**A**) UV–visible spectra. (**B**) Energy bandgap of (a) g-C_3_N_4_, (b) HAp and (c) HAp/g-C_3_N_4_ nanocomposites.

To investigate the optical absorption behavior of the g-C_3_N_4_, HAp and HAp/g-C_3_N_4_ nanocomposites are confirmed through UV–visible spectroscopy and the corresponding results are shown in Fig. 10A (a–c). In Fig. 10A (a), HAp nanospheres have a very low absorption capacity in the UV-light region. The obtained optical absorption here are similar to those reported in the previous article^30^. The absorption edge of the g-C_3_N_4_ nanosheets is observed at 470 nm. It is capable of wider and stronger absorption in both the UV and visible light regions. The HAp/g-C_3_N_4_ nanocomposite exhibit absorption edges are similar to g-C_3_N_4_ nanosheets. When adding HAp nanosphere to g-C_3_N_4_ nanosheets the absorption intensity was increased. The g-C_3_N_4_ nanosheets attaching to the surface of the HAp nanospheres result in enhanced light-harvesting absorption in both the UV and visible regions. The energy bandgap of the g-C_3_N_4_, HAp and HAp/g-C_3_N_4_ nanocomposite were calculated using the Tauc plot^31^ and the corresponding outcomes are displayed in Fig. 10B. The calculated energy bandgap of g-C_3_N_4_, HAp and HAp/g-C_3_N_4_ nanocomposite were found to be 2.71, 4.6, and 2.59 eV, respectively. A decrease in the energy bandgap could be attributed to strong interactions between HAp and g-C_3_N_4_ nanosheets. The combination of these two materials creates a greater number of charge separation states. Based on the outcomes, high light absorption capacity and suitable energy bandgap make these materials suitable as photocatalysts in environmental applications.

**Figure 11:**
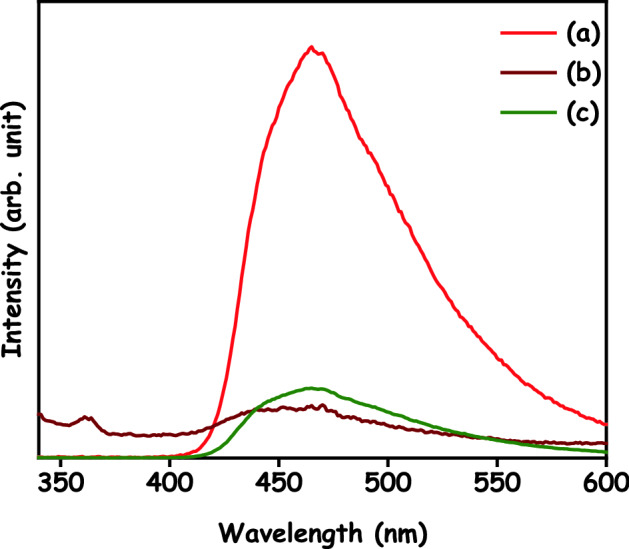
PL spectra of (a) g-C_3_N_4_, (b) HAp and (c) HAp/g-C_3_N_4_ nanocomposites.

To demonstrate the separation efficiency of the photogenerated electron–hole pairs in the photocatalytic process, PL spectra were engaged at a suitable excitation wavelength. As shown in Fig. 11, PL spectra of g-C_3_N_4_, HAp and HAp/g-C_3_N_4_ nanocomposite were recorded using an excitation wavelength of 350 nm at room temperature. The main peak of pure g-C_3_N_4_ emission was detected at 464 nm, in match with the previous article^32^. This strong peak in the spectrum can be attributed to the energy bandgap recombination of e^–^h^+^ pairs. Generally, a higher PL emission intensity was acceptable to indicate faster recombination of photogenerated e^–^h^+^ pairs. A lower PL emission intensity designates that a greater number of electrons are being transferred or trapped. In this connection, the HAp did not affect the position of the emission peak, but rather reduced its emission intensity relative to pure g-C_3_N_4_. The decreased emission intensity indicates that photoexcited electrons can be efficiently transferred between HAp and g-C_3_N_4_. Due to the extended lifetime of e^−^ and h^+^ in the excited state, higher quantum efficiency can be reached, which consequences in enriched photocatalytic performance against organic contaminates.

### Photocatalytic degradation of MB dye and doxycycline drug

**Figure 12:**
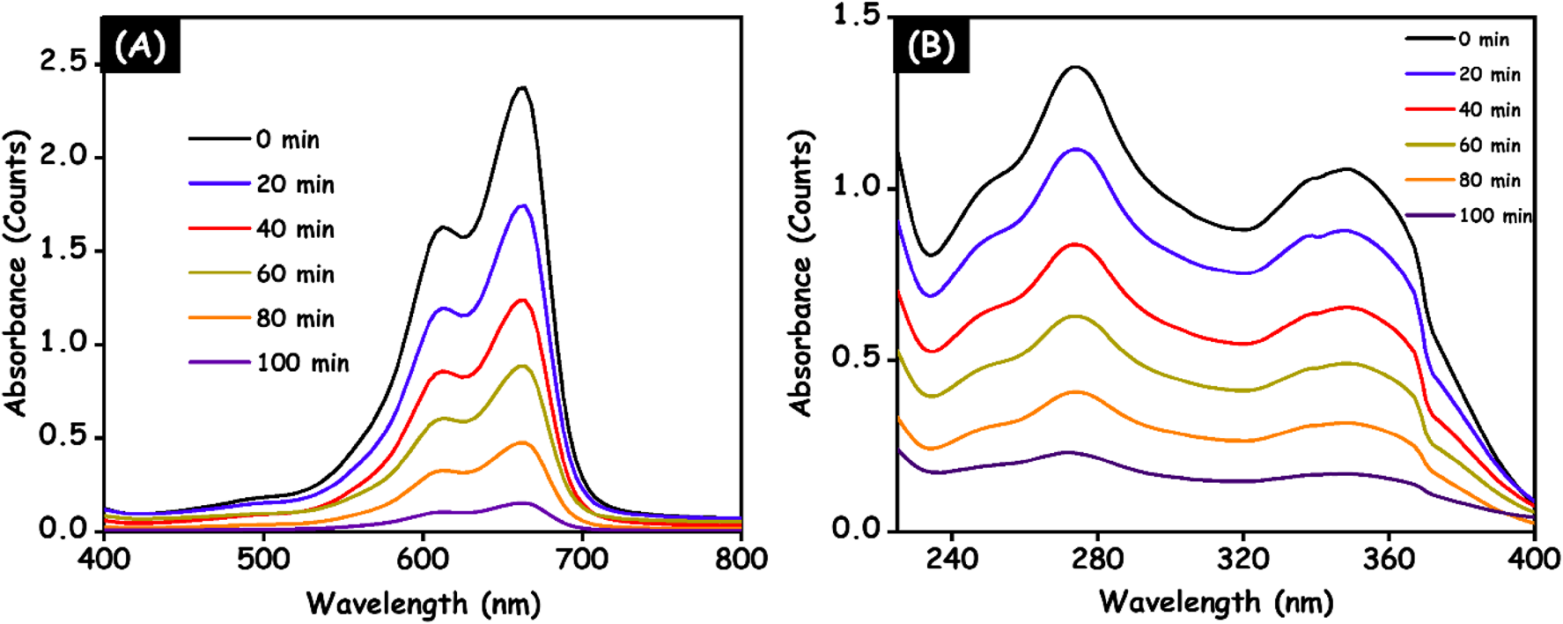
Photocatalytic performance of HAp/g-C_3_N_4_ nanocomposite evaluated against (**A**) MB dye and (**B**) doxycycline drug.

Under the visible light illumination, the photocatalytic degradation ability of the g-C_3_N_4_, HAp and HAp/g-C_3_N_4_ nanocomposite were investigated against the degradation of MB dye and doxycycline drug and the corresponding outcomes are displayed in Fig. 12A,B. All the prepared samples exhibit low absorbability after 20 min in dark condition. Figure 12A, the maximum absorption peak of MB dye was observed at 663 nm and Fig. 12B clearly indicates that, the maximum absorption peak of the doxycycline drug was observed at 270 and 352 nm, which is suitable promise with earlier reported articles^33^. In 100 min of visible light illumination, the HAp/g-C_3_N_4_ nanocomposite showed excellent degradation efficiency than pure samples under UV–visible light irradiation for both MB dye and doxycycline drug. The observed photocatalytic degradation results from Fig. 12A,B clearly indicate that the addition of HAp with g-C_3_N_4_ improves the degradation performance. Compared with pure the g-C_3_N_4_ and HAp, the HAp/g-C_3_N_4_ nanocomposite showed much higher photocatalytic degradation ability which may be due to the enhanced visible light absorption and synergetic effect of the prepared sample.

**Figure 13:**
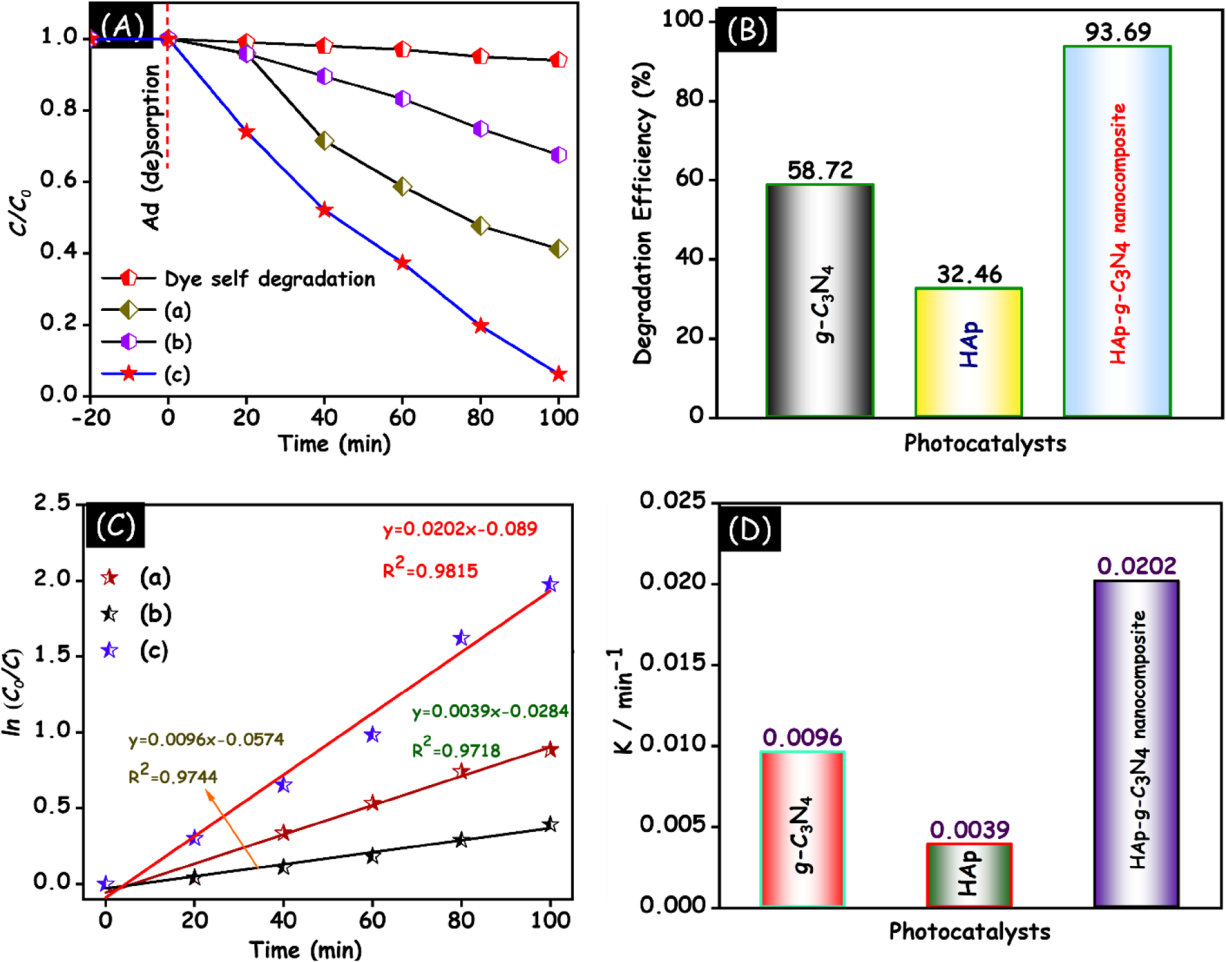
(**A**) Photocatalytic performance. (**B**) Degradation efficiency. (**C**) Pseudo first order kinetic model of (a) g-C_3_N_4_, (b) HAp and (c) HAp/g-C_3_N_4_ nanocomposites and (**D**) rate constant of HAp/g-C_3_N_4_ nanocomposite for MB dye degradation under UV–visible light irradiation.

In Fig. 13A, the self-degradation of MB dye was investigated and the outcomes indicated that the MB dye cannot degrade by itself under UV–visible light irradiation. From Fig. 13B, the photodegradation efficiency of MB dye over g-C_3_N_4_, HAp and HAp/g-C_3_N_4_ nanocomposite was found to be 58.72, 32.46 and 93.69%, respectively. To better understand the photocatalytic performance of MB degradation reaction by different photocatalysts, the kinetic of MB dye was fitted to the pseudo-first-order kinetic model. Figure 13C shows the linear relationship between time and ln (C/C_0_). The correlation coefficient of g-C_3_N_4_, HAp and HAp/g-C_3_N_4_ nanocomposite are determined to be 0.9744, 0.9718 and 0.9815, respectively. According to the outcome results, all prepared samples are linearly fit, and their R^2^ values are nearly 1. The bar graph in Fig. 13D signifies the comparative rate constant for all the prepared samples.

**Figure 14:**
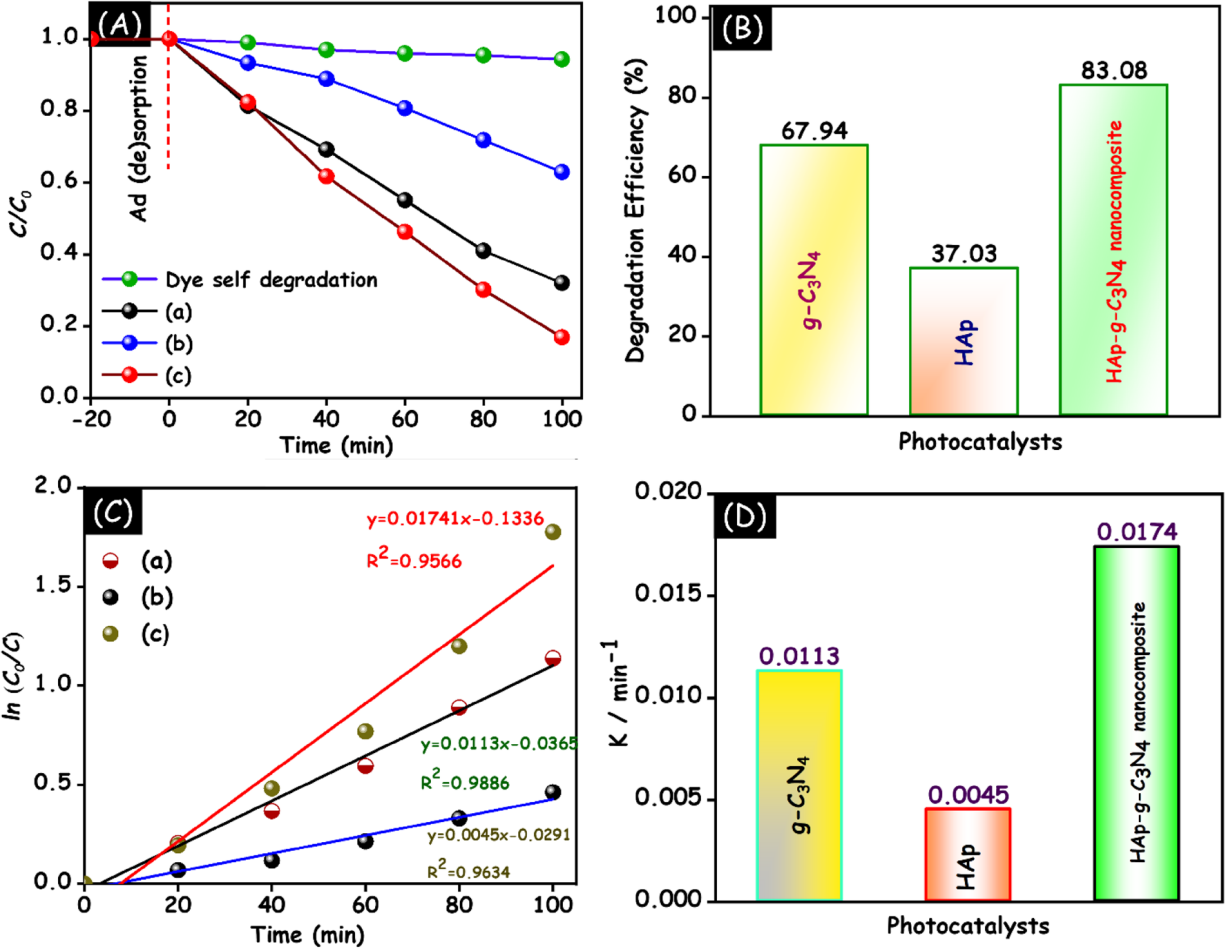
(**A**) Photocatalytic performance. (**B**) Degradation efficiency. (**C**) Pseudo first order kinetic model of (a) g-C_3_N_4_, (b) HAp and (c) HAp/g-C_3_N_4_ nanocomposites and (**D**) rate constant of HAp/g-C_3_N_4_ nanocomposite for doxycycline dye degradation under UV–visible light irradiation.

As shown in Fig. 14A, a similar MB dye degradation process was utilized to investigate the doxycycline drug eviction in the presence of as-prepared samples under UV–visible light irradiation. In Fig. 14B, the photodegradation efficiency of doxycycline drug over g-C_3_N_4_, HAp and HAp/g-C_3_N_4_ nanocomposite is 67.94, 37.03, and 83.08% respectively. To confirm the quantitative confirmation of doxycycline degradation, the pseudo first-order kinetic model was employed. Figure 14C demonstrates the as prepared HAp/g-C_3_N_4_ nanocomposite exhibit good linear performance and is well fit in the pseudo first order kinetic model. The correlation coefficient of the g-C_3_N_4_, HAp and HAp/g-C_3_N_4_ nanocomposite are 0.9634, 0.9886 and 0.9815 respectively. The rate constant (bar graph) of the prepared samples is visualized in Fig. 14D. The rate constant follows the order of HAp/g-C_3_N_4_ nanocomposite > g-C_3_N_4_ > HAp, respectively. Based on the above results, it is clearly seen that, HAp/g-C_3_N_4_ nanocomposite exhibit superior photocatalytic performance on doxycycline drug degradation due to the interfacial contact between g-C_3_N_4_ nanosheets and HAp nanoparticles.

**Figure 15:**
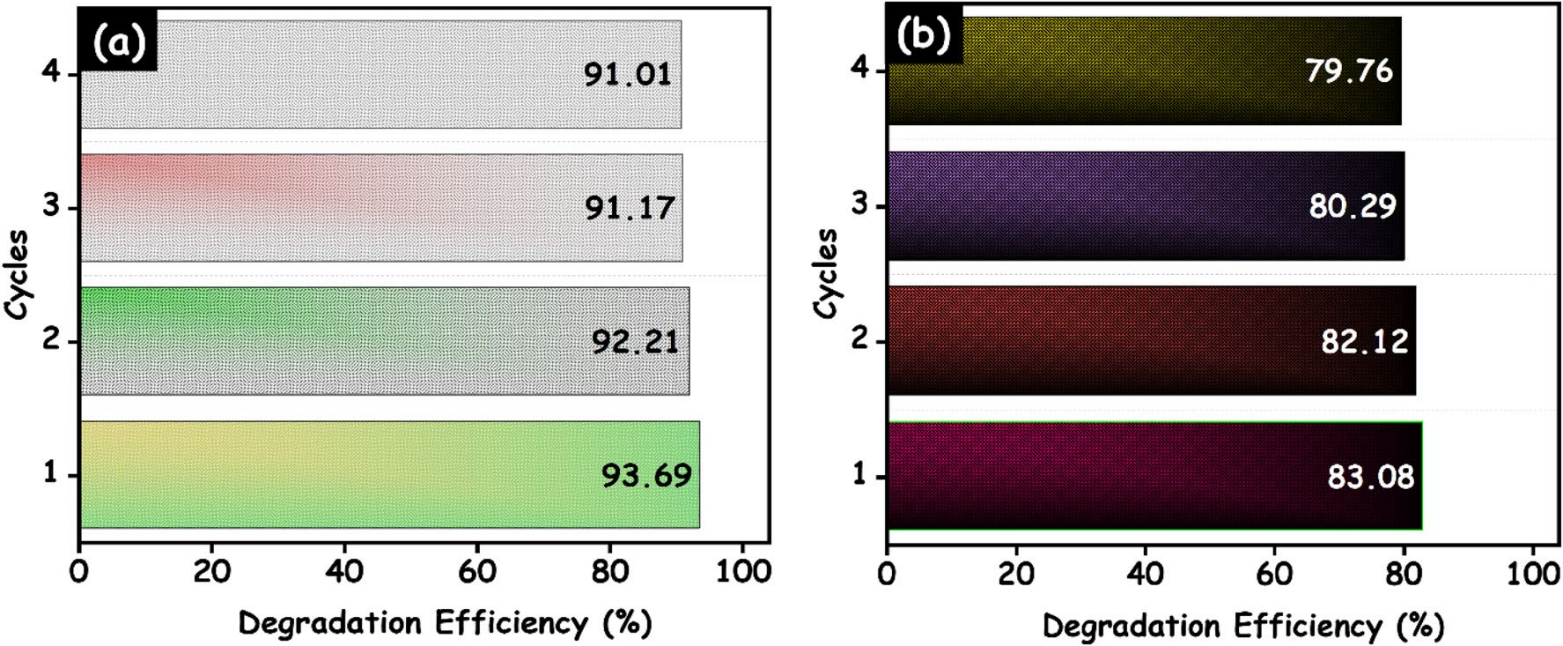
Recycle runs for the photocatalytic degradation of (**a**) MB and (**b**) doxycycline drug over HAp/g-C_3_N_4_ nanocomposite under UV–visible light irradiation.

The stability and reuse performance of the materials is an important parameter and cost reduction of the treatment from the practical application point of view. The recycling performance of the HAp/g-C_3_N_4_ nanocomposite is carried out in four consecutive cycles through a similar process of photocatalytic activity. Figure 15A,B shows the photostability of a HAp/g-C_3_N_4_ nanocomposite for both the MB dye and doxycycline drug. Each cycle was run for 100 min, afterward each run the reaction photocatalyst was collected and washed. The photocatalytic degradation efficiency of MB dye decreased from 93.69 to 91.01% while that of doxycycline drugs decreased from 83.08 to 79.76%, respectively. This decrease in efficiency may have occurred due to the loss of photocatalyst during the recollecting process and also to the small size of fragmented species attached to the photocatalyst materials.

**Figure 16:**
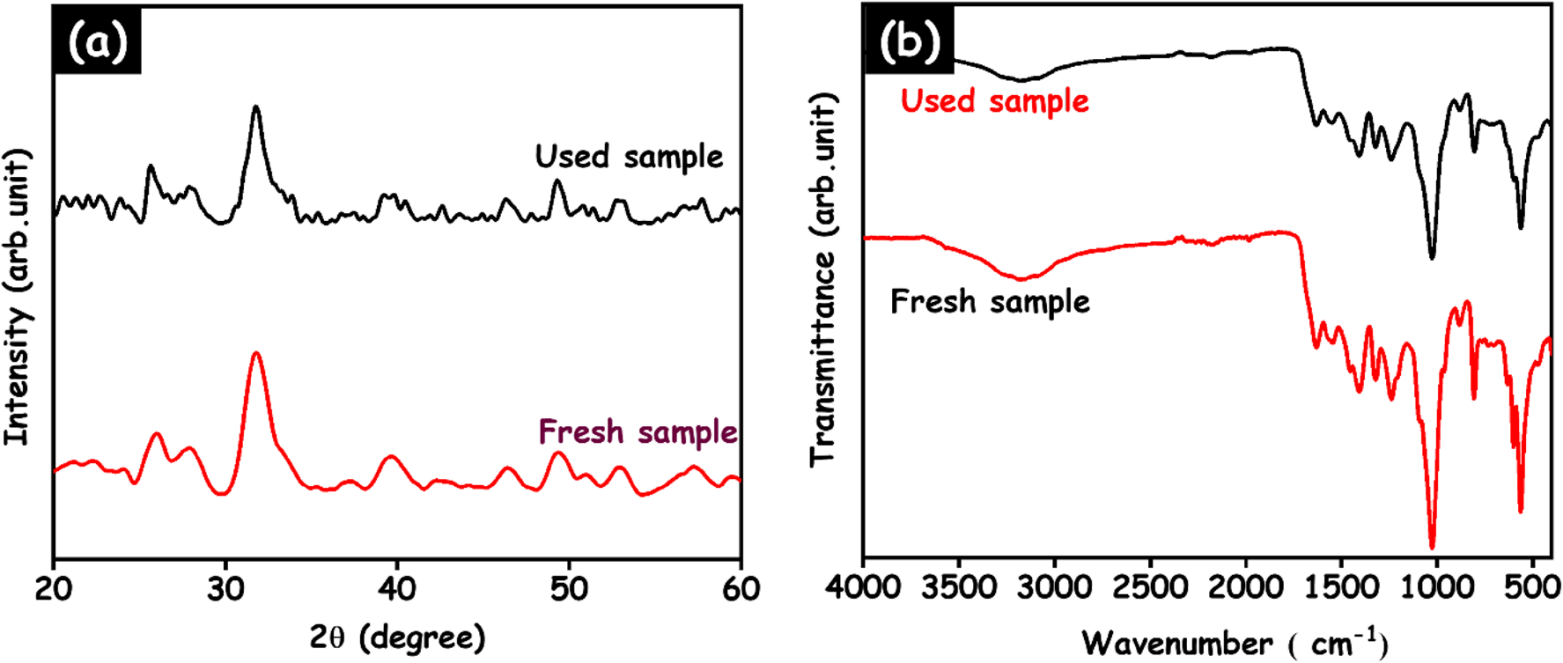
(**a**) XRD patterns and (**b**) FTIR spectra for HAp/g-C_3_N_4_ nanocomposite fresh and used 4 cyclic runs.

XRD and FTIR analysis was conducted after the cycling process to assess the stability of the HAp/g-C_3_N_4_ nanocomposite and the corresponding consequences are demonstrated in Fig. 16a,b. In Fig. 16a, the structural behaviour of the HAp/g-C_3_N_4_ nanocomposite clearly shows that there are no additional diffraction peaks in the fourth run. The functional groups and vibration peaks also remained unchanged to confirm in the FTIR analysis. Based on these results, it is confirmed that the HAp/g-C_3_N_4_ nanocomposite materials have high stability. The photocatalytic performance of the HAp/g-C_3_N_4_ nanocomposite as compared to recently published articles and the consequences are revealed in Table 1.

**Table 1:** Comparison of photocatalytic efficiency of HAp/g-C_3_N_4_ nanocomposite with various photocatalysts for MB dye and doxycycline drug.

S. no	Samples	Organic dyes	Time (mins)	Efficiency (%)	Reference
1	CeO_2_/V_2_O_5_	MB	300	~ 76.8	^34^
2	Graphite/TiO_2_	MB	240	~ 85	^35^
3	g-C_3_N_4_ /TiO_2_ nanocomposite	MB	156	~ 90	^36^
4	g-C_3_N_4_ thin layer @ CeO_2_ nanocomposite	Doxycycline drug	60	~ 84	^7^
5	Cerium doped zinc aluminate	MB	240	~ 72.5	^37^
6	titanium-doped hydroxyapatite	MB	240	~ 93	^38^
7	Ag_3_PO_4_/HAp@γ-Fe_2_O_3_ nanocomposite	MB	240	~ 99	^16^
8	ZnO nanoparticles	Doxycycline drug	300	~ 99	^33^
9	Cu_2_O/BiVO_4_ nanocomposite	MB	160	~ 72.9	^39^
10	HAp/g-C_3_N_4_ nanocomposite	MB	100	93.69	This work
		Doxycycline drug	100	83.08	

### Reaction mechanism

**Figure 17:**
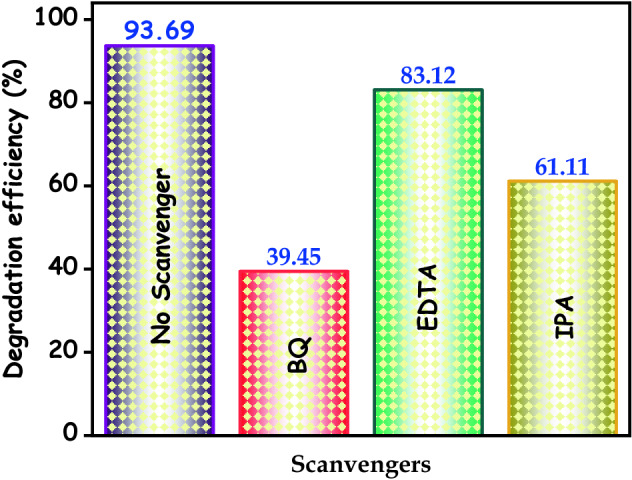
Scavenger test during the degradation of MB over HAp/g-C_3_N_4_ nanocomposite under UV visible light irradiation.

To understand the photodegradation mechanism of the HAp/g-C_3_N_4_ nanocomposite, scavenger experiments were employed. To investigate which active species are very important in the photocatalytic degradation process. Generally, the lesser degradation efficiency in the degradation system indicates the presence of major active species. From Fig. 17, it is clearly noted that the BQ scavenger was added and the photocatalytic performance of the HAp/g-C_3_N_4_ nanocomposite was fully suppressed. These results indicate that the superoxide radical is a chief role in the degradation process of HAp/g-C_3_N_4_ nanocomposite. The photocatalytic degradation efficiency was weakly decreased and small changes in both EDTA and IPA scavengers, which mean the hydroxyl and hole scavengers play a minor role in the degradation process. The scavenger test clearly confirmed that the strong contribution of superoxide radicals plays an important role in the photocatalytic system. These superoxide radicals will be reduce a large number of surface oxygen species.

**Figure 18:**
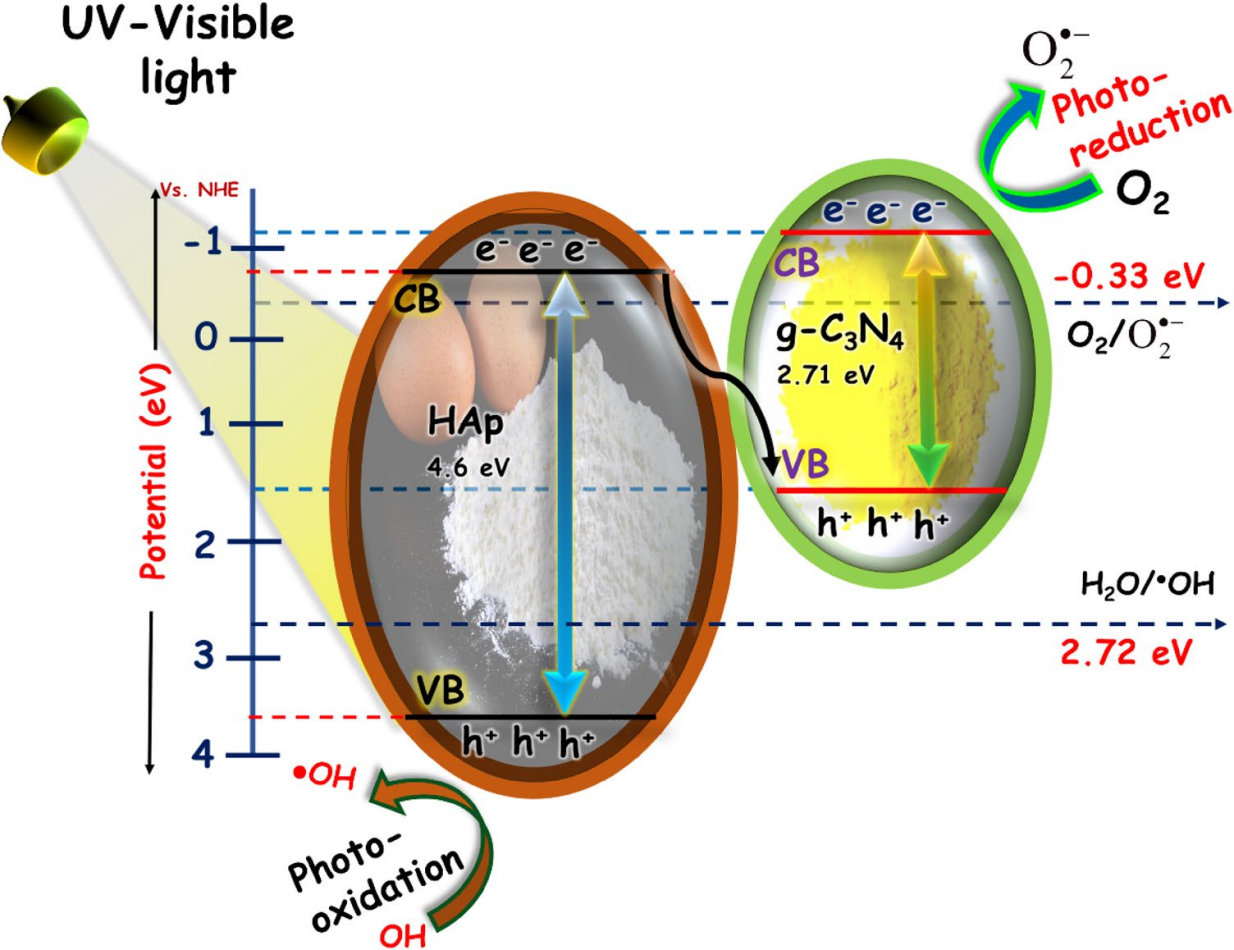
Proposed direct Z-scheme photocatalytic mechanism of the HAp/g-C_3_N_4_ nanocomposite under UV–Visible light irradiation.

The possible photocatalytic mechanism of the HAp/g-C_3_N_4_ nanocomposite are shown in Fig. 18. The valence and conduction band edge position of the HAp/g-C_3_N_4_ nanocomposite is a very important discussion of the photogenerated charge route. The band energy position of the HAp and g-C_3_N_4_ nanosheet are calculated using Mullikan electronegativity theory^40^. The absolute electronegativity of the HAp and g-C_3_N_4_ nanosheets are 5.89^41^ and 4.72 eV^42^, respectively. The calculated conduction and valence band of the HAp and g-C_3_N_4_ nanosheets are − 0.91, − 1.135 eV and 3.69, 1.575 eV respectively. On the other hand, the energy bandgap of the HAp and g-C_3_N_4_ nanosheets are estimated to be 4.6 and 2.71 eV, respectively. When the light irradiates the HAp/g-C_3_N_4_ nanocomposite, the electron moves from VB of HAp to conduction band of HAp. So, the CB electrons of HAp are easily transferred to VB of g-C_3_N_4_ nanosheets. The g-C_3_N_4_ nanosheets CB position value was more negative than the standard potential (E(O_2_/^·^O_2_^−^ = − 0.33 V))^43^. Thus, electrons are easily reacted with surface O_2_ molecules to produce the superoxide radicals on the g-C_3_N_4_ nanosheets. Meanwhile, the VB value of g-C_3_N_4_ nanosheets (1.575 eV) is more negative than that of the standard potential (E(^·^OH/H_2_O) = 2.27 eV), which makes it impossible to produce a greater number of hydroxyl radicals. Nevertheless, the VB potential of HAp (3.69 eV) was larger than that of g-C_3_N_4_ nanosheets, so the photogenerated holes react with H_2_O to produce as many hydroxyl radicals as possible. Based on the scavenger results, it is clearly confirmed that the superoxide and hydroxyl radicals play a major role in the photocatalytic performance of MB dye and pharmacological drugs. Consequently, the migration and transformation of the HAp/g-C_3_N_4_ nanocomposite Z-scheme heterojunction not only expands the light absorption range of HAp, but also maintains the electron and holes with higher reduction and oxidation potential. Thus, a peculiar band structure achieves the enriched photocatalytic performance of MB dye and doxycycline drug in aqueous medium. The detailed process of the HAp/g-C_3_N_4_ nanocomposite photocatalytic performance is given by Eqs. (1–7)

1 $${\text{HAp}} + {\text{g - C}}_{{3}} {\text{N}}_{{4}} \to {\text{electron (e}}^{ - } {\text{) + holes (h}}^{ + } {)}$$

2 $${\text{electrons (e}}^{ - } {\text{) + surface oxygen molecules (O}}_{{2}} ) \to {\text{superoxide radicals (}}^{ \bullet } {\text{O}}_{2}^{ - } )$$

3 $${\text{holes (h}}^{ + } {\text{) + water (H}}_{{2}} {\text{O}}) \to {\text{hydroxyl radicals (}}^{ \bullet } {\text{OH}})$$

4 $$^{ \bullet } {\text{OH + MB dye}} \to {\text{ products}}$$

5 $$^{ \bullet } {\text{OH + doxycycline drug}} \to {\text{ products}}$$

6 $$^{ \bullet } {\text{O}}_{2}^{ - } {\text{ + doxycycline drug}} \to {\text{ H}}_{{2}} {\text{O + CO}}_{{2}} {\text{ + etc}}{.,}$$

7 $$^{ \bullet } {\text{O}}_{2}^{ - } {\text{ + MB dye}} \to {\text{ H}}_{{2}} {\text{O + CO}}_{{2}} {\text{ + etc}}{.,}$$

The observed electron path agrees well with the Z-scheme mechanism, which could enrich the charge carrier separation and keep the strong redox potential to capably eliminate the dangerous dyes and pharmacological drugs.

## Conclusion

In summary, Z-scheme based eggshell derived HAp/g-C_3_N_4_ nanocomposite was successfully fabricated through a simple precipitation technique. The HAp/g-C_3_N_4_ nanocomposite displays outstanding photodegradation ability compared to the bare HAp and g-C_3_N_4_ samples. With proper band alignment and effective visible light absorption, the HAp/g-C_3_N_4_ nanocomposite efficiently removed MB dye (93.69%) and doxycycline drugs (83.08%) within 100 min. The HAp/gC_3_N_4_ photocatalyst exhibits a rate constant higher than 5.17 times that of HAp and 2.14 times that of g-C_3_N_4_ nanosheets, respectively. In the recycling experiment, HAp/g-C_3_N_4_ nanocomposite shows excellent photostability and reusability performance. It has also been noted that superoxide and hydroxyl radicals play a major role in the breakdown of hazardous contaminants. Therefore, this work confirms that the biowaste (eggshell) derived HAp embedded on g-C_3_N_4_ nanosheets exhibits promising photocatalyst materials for an eco-friendly degradation process.

## Data Availability

The datasets used and/or analysed during the current study available from the corresponding author on reasonable request.
